# Systematic review of the effectiveness of training programs in writing for scholarly publication, journal editing, and manuscript peer review (protocol)

**DOI:** 10.1186/2046-4053-2-41

**Published:** 2013-06-17

**Authors:** James Galipeau, David Moher, Becky Skidmore, Craig Campbell, Paul Hendry, D William Cameron, Paul C Hébert, Anita Palepu

**Affiliations:** 1 Ottawa Hospital Research Institute, 501 Smyth Rd, Ottawa, K1H 8L6, Canada; 2 Faculty of Medicine, University of Ottawa, 451 Smyth Road, Ottawa, ON, K1H 8M5, Canada; 3 Independent Consultant, Ottawa, Canada; 4 Royal College of Physicians and Surgeons of Canada, 774 Echo Drive, Ottawa, ON, K1S 5N8, Canada; 5 Department of Medicine, Centre for Health Evaluation and Outcome Sciences, University of British Columbia, St Paul’s Hospital, Vancouver, BC, V6Z 1Y6, Canada

**Keywords:** Training, Writing for publication, Journalology, Author, Journal editor, Manuscript peer review, Publishing

## Abstract

**Background:**

An estimated $100 billion is lost to ‘waste’ in biomedical research globally, annually, much of which comes from the poor quality of published research. One area of waste involves bias in reporting research, which compromises the usability of published reports. In response, there has been an upsurge in interest and research in the scientific process of writing, editing, peer reviewing, and publishing (that is, journalology) of biomedical research. One reason for bias in reporting and the problem of unusable reports could be due to authors lacking knowledge or engaging in questionable practices while designing, conducting, or reporting their research. Another might be that the peer review process for journal publication has serious flaws, including possibly being ineffective, and having poorly trained and poorly motivated reviewers. Similarly, many journal editors have limited knowledge related to publication ethics. This can ultimately have a negative impact on the healthcare system. There have been repeated calls for better, more numerous training opportunities in writing for publication, peer review, and publishing. However, little research has taken stock of journalology training opportunities or evaluations of their effectiveness.

**Methods:**

We will conduct a systematic review to synthesize studies that evaluate the effectiveness of training programs in journalology. A comprehensive three-phase search approach will be employed to identify evaluations of training opportunities, involving: 1) forward-searching using the Scopus citation database, 2) a search of the MEDLINE In-Process and Non-Indexed Citations, MEDLINE, Embase, ERIC, and PsycINFO databases, as well as the databases of the Cochrane Library, and 3) a grey literature search.

**Discussion:**

This project aims to provide evidence to help guide the journalological training of authors, peer reviewers, and editors. While there is ample evidence that many members of these groups are not getting the necessary training needed to excel at their respective journalology-related tasks, little is known about the characteristics of existing training opportunities, including their effectiveness. The proposed systematic review will provide evidence regarding the effectiveness of training, therefore giving potential trainees, course designers, and decision-makers evidence to help inform their choices and policies regarding the merits of specific training opportunities or types of training.

## Background

An estimated $100 billion is lost to ‘waste’ in biomedical research globally each year, a sizeable portion of which comes from the poor quality of published research. Chalmers and Glasziou identified four areas of waste related to: 1) the relevancy of research questions to clinicians and patients, 2) the appropriateness of research design and methods, 3) making publications fully accessible, and 4) issues of bias and the usability of reports [1]. In the latter of these categories, the authors explain that over 30% of trial interventions are not sufficiently described, over 50% of planned study outcomes are not reported and most new research is not interpreted in the context of systematic assessment of other relevant evidence [2]. In response to this, there has been an upsurge in interest and research on topics such as publication ethics, research integrity, and rigor in the scientific process of writing, editing, peer reviewing, and publishing (that is, journalology) of biomedical research. This type of waste has also become a primary focus of organizations such as the Committee on Publication Ethics (COPE), World Association of Medical Editors (WAME), and the Enhancing the Quality and Transparency of Health Research (EQUATOR) Network.

Bias in reporting and the problem of unusable reports can be attributed to shortcomings at both the production and publication phases of the research process. On one hand, some authors lack knowledge or engage in questionable practices while designing, conducting, or reporting their research. On the other hand, the peer review process for both grant giving and journal publication has serious flaws, including claims of being ineffective [3], as well as having poorly trained and poorly motivated reviewers. Similarly, many journal editors lack formal training [4,5], as well as having poor knowledge related to publication ethics [5]. While the causes for this type of research waste may be varied, the consequences for decision-makers, knowledge users, and tax-paying healthcare patients are ultimately negative, as indicated by Dickerson and Chalmers in their 2010 report on this topic: ‘Incomplete and biased reporting has resulted in patients suffering and dying unnecessarily [6]. Reliance on an incomplete evidence base for decision-making can lead to imprecise or incorrect conclusions about an intervention’s effects. Biased reporting of clinical research can result in overestimates of beneficial effects [7] and suppression of harmful effects of treatments [8]. Furthermore, planners of new research are unable to benefit from all relevant past research [9]’.

The lack of formal training appears to be widespread not only among authors of health research, but also among the gatekeepers of health literature - journal peer reviewers and editors, and at earlier stages, grant peer reviewers. This may be one potential reason for the large amount of waste in biomedical research. Murray [10] suggests that most academics have no formal training in writing for publication and that they developed their skills mainly through a process of trial and error. In addition, the rates of author misconduct [11-14], of which most incidences stem from negligence, poorly performed science, investigator bias, or lack of knowledge, rather than acts of fraud [15], suggest a need for better training among authors on journalological issues. Meanwhile, Keen [16] argued that, while there is a wealth of literature describing how to go about writing for publication, the provision of information alone may be insufficient to support potential authors. In addition, Eastwood [17] suggested that professional training opportunities may be lacking due to a faulty assumption that trainees could not have achieved their postdoctoral status without having acquired an education in critical reading and writing.

In the case of peer reviewers, very little is known about their training and experiences [18], however, research shows that many reviewers’ needs for training and support are not being met, despite the desire for it among most of them [19]. Peer reviewers have difficulty identifying major errors in articles submitted for publication [20-22] and in some cases agreement between reviewers of the same manuscript is not much different than would be expected by chance [3]. There is also evidence to suggest that the quality of one’s peer reviewing deteriorates over time [3,18] and that peer reviewers are susceptible to positive-outcome bias [23]. Similarly, the peer review process used by granting agencies also appears to be problematic. A survey of 29 international granting agencies indicated that several aspects of their peer review process were poor and had not improved in the preceding 5 years, including difficulty retaining good reviewers, reviewers carrying out poor quality reviews, and reviewers not following guidelines appropriately [24]. The same survey polled external reviewers of granting agencies (n = 258) and found that only 9% had received some form of training in how to conduct biomedical grant reviews, despite 64% of reviewers expressing an interest in peer review training [24]. The authors concluded by saying that funding organizations should help reviewers do their job effectively by providing clear guidance and training.

Many journal editors report having informal [5], or little to no [4] training in editing skills, as well as being unfamiliar with available guidelines [25]. However, they also say they would welcome more guidance or training [4,5,25]. When tested, editors performed very poorly on knowledge of editorial issues related to authorship, conflict of interest, peer review, and plagiarism [5]. Many editors also believed that ethical issues occur rarely or never at their journal [25]. These findings echo the assertion of Paul Hébert, former Editor-in-Chief of the *Canadian Medical Association Journal* (CMAJ), that ‘we need to train the editors of tomorrow. In Canada, we have a very small scholarly publishing industry. As a consequence, there are few medical editing positions, no obvious career paths and even fewer training opportunities [26]’.

While the reasons for the poor training of authors, peer reviewers, and editors have not been studied directly, one cause may be a lack of legitimate opportunities to obtain formal training [26], or a lack of access to these training opportunities. For example, there are currently no certification programs or degrees that allow a physician to train specifically to become a medical journal editor. While courses are offered by a few groups (for example, Council of Science Editors) and Fellowship training programs exist in the USA, Canada, and the UK, the majority of these are 1-year programs, largely require a full-time commitment, and are accessible to only a select few, annually. Some journals, such as the *Journal of the American Medical Association* (JAMA), the *British Medical Journal* (BMJ) and *American Family Physician* (AFP) have created 1- to 2-month electives for medical students to undergo similar training; however, these are only open to medical students. The situation for authors and peer reviewers is not much better. Eastwood [17] explains that, for medical residents, journal clubs are the primary forum in which to learn about critical appraisal of biomedical literature, however, most clinicians will receive little [27] to no [10] formal training in writing for publication. Eastwood also points out that ‘few of the programs developed to meet the National Institutes of Health requirement for training in responsible research practices devote time to the practice and ethics of biomedical reporting [17]’. Similarly, for peer reviewers, there is little to no formal training available, with most reviewers being guided by journals’ instructions to reviewers sections and being forced to learn by trial-and-error [28].

There have been repeated calls for better, more numerous training opportunities for research reporting, peer review, and publishing [1,26,29]. Although training opportunities appear to be somewhat limited, a small number of them do exist, some being offered by reputable organizations. However, little research has taken stock of the journalology training opportunities or related evaluations of their effectiveness. A systematic review of training opportunities in a related area - overcoming barriers to publication - has been identified [30]. The review, which included 17 studies published between 1984 and 2004, evaluated the effect of writing courses, writing support groups and writing coaches on author output. While publication rates were found to increase overall, whether or not opportunities exist to enhance the quality of such research output for all relevant players (that is, authors, peer reviewers and editors), is a vastly more relevant question in the age of increasing evidence towards author misconduct and misreporting. We are not aware of an existing synthesis of knowledge on this topic.

The objective of this project is to systematically review, evaluate and synthesize information on whether training in journalology effectively improves educational outcomes, such as measures of knowledge, intention to change behaviour, and measures of excellence in training domains. Collecting this information will allow knowledge users to know what training options are most effective. This will be useful for making training recommendations to authors, peer reviewers, editors, and others. In addition, it will provide a solid foundation from which to develop and build new training courses and programs for these groups, ultimately improving knowledge and the quality of research practices both within Canada and abroad.

## Methods

### Criteria for considering studies for this review

#### *Population*

Those centrally involved in writing for scholarly publication, journal editing, and manuscript peer review (that is, authors, peer reviewers, journal editors), or any other group that may be peripherally involved in the scientific writing and publishing process, such as medical journalists.

#### *Intervention*

Evaluations of training in any specialty or subspecialty of writing for scholarly publication, journal editing, or manuscript peer review targeted at the designated population(s) will be included.

#### *Comparator*

The following comparisons will be included: 1) before and after administration of a training class/course/program of interest, 2) between two or more training classes/courses/programs of interest, or 3) between a training class/course/program and any other intervention(s) (including no intervention).

#### *Outcome(s)*

The primary outcomes will be any measure of effectiveness of training as reported, including, but not limited to: measures of knowledge, intention to change behaviour, measures of excellence in training domains (writing, peer review, editing), however reported. Since this review is largely exploratory, where other meaningful outcomes are reported, this information will be collected as well.

#### *Study design(s)*

Comparative studies evaluating at least one training program/course/class of interest will be included in this review and henceforth be termed ‘evaluations’.

#### *Search methods for identification of studies*

A comprehensive three-phase search approach will be employed to identify evaluations of training opportunities, as follows: 1) For training which has been described in published reports, citations of these reports will be forward-searched using the Scopus citation database. 2) We will also perform a search of MEDLINE In-Process and Non-Indexed Citations, MEDLINE, Embase, ERIC, PsycINFO, and the databases of the Cochrane Library. A specific search strategy will be developed by an information specialist and will be peer reviewed prior to execution [31]. There will be no language restrictions on the search strategy, however, due to the large expected yield of the planned review and limited resources available, evaluations encountered in languages other than English and French will be set aside and listed in an appendix in the report. Letters, commentaries and editorials will not be excluded due to the possibility that they may contain reference to evaluations of particular training programs. Studies will not be excluded based on publication status. 3) A grey literature search will also be conducted, consisting of screening the results of a concurrent project to map all existing and previous training in journalology through an ‘environmental scanning’ technique [32,33] using the Google search engine. The administrators of any relevant training opportunities identified in the environmental scan will be contacted to inquire whether they are aware of any published or unpublished evaluations of the training opportunities.

#### *Data collection*

Following the execution of the search strategy, the identified records (titles and/or available abstracts) will be collated in a Reference Manager [34] database for de-duplication. The final unique record set and full-text of potentially eligible studies will be exported to an Internet-based software, DistillerSR (Evidence Partners, Ottawa, Canada), through which screening of records and extraction of data from included evaluations will be carried out.

#### *Study selection*

Given the broad/general nature of many of the search terms (for example, author, editor, education) we expect a large volume of initial search results. Therefore, we will conduct an initial screening of the titles only by two reviewers, using the liberal acceleration method (that is, one reviewer screens all identified studies and a second reviewer screens only excluded studies, independently). Following the title screen, titles and abstracts of identified records will be screened by two reviewers, again using the liberal accelerated method. Subsequently, the full-text of all potentially eligible evaluations will be retrieved and reviewed for eligibility, independently, by two members of the team using *a priori* eligibility criteria. To be included, evaluations must include one of the *a priori* comparison groups and examine the influence of training, as reported, using any measure. Disagreements between reviewers at this stage will be resolved by consensus or by a third member of the research team. If necessary, authors of potentially included evaluations will be contacted to clarify data needed for eligibility.

#### *Data extraction*

Separate data extraction forms will be developed to capture information needed for synthesis for each of the three comparisons and will be piloted using a subset of included evaluations and modified, as needed. One reviewer will extract general study characteristics of included evaluations, with verification carried out by a second reviewer. Data on measures of effectiveness of training for each program/course/class will be extracted by one reviewer; a second reviewer will verify the accuracy of the data from a random 20% sample of included evaluations. Any discrepancies between reviewers will be resolved by consensus or by a third member of the research team. If there is greater than a 50% discrepancy between reviewers’ answers in the random 20% sample, or only a small number of included studies, 100% data verification will be considered. Authors of included evaluations will be contacted to invite contribution of any unpublished data needed for this review that is not available in published reports.

General publication characteristics to be extracted include: first author name and contact information (of first or corresponding author), year of publication, institutional affiliation of first author, country, language of publication, type of document (full text versus abstract), and funding source. Other details to be collected include: name of training class(es)/course(s)/program(s) being evaluated (if applicable), population evaluated, sample size, whether prospective or retrospective, and mechanism of sampling (or participant assignment to groups). Extracted outcome data will include: tool(s) used to evaluate effectiveness of training, timing of measurement, effectiveness measurement (however reported), intention to change behaviour scores, and measures of excellence data (however reported).

#### *Assessment of validity of evaluations*

No tool currently exists to assess the validity (internal and external) of evaluations in methodological reviews such as this. Study designs are expected to be largely heterogeneous, however, if evaluations using randomized control trial (RCT) designs are encountered, the Cochrane risk of bias tool will be used to judge validity [35]. To assess all other evaluations, we propose assessing the following criterion with a rating of ‘yes’, ‘no’ or ‘unclear’, to help readers make their own judgments about the overall validity of the included evidence. We have used this approach elsewhere [36,37].

1. Whether an objective measure of training effectiveness was employed (that is, *a priori* questionnaires).

2. Whether the measurement tool to evaluate training effectiveness was reported to be validated.

3. Whether intended methods align with reported findings.

4. Whether data from all included participants was reported.

5. Whether sampling for comparison 2. and 3. occurred within the same time frame.

6. Whether comparison groups represent similar populations (that is, same area of health-related discipline, similar levels of training).

### Data analysis

#### *Measures of effect*

Due to the paucity of literature describing formal training opportunities in journalology, we are unable to anticipate the types of measurement tools that might be used for their evaluation. Where data is provided narratively, it will be collected as such. Where summary scores of outcomes (that is, participant knowledge using a particular tool) are presented within each evaluation, we will collect means and standard deviations (SDs). When medians and ranges are reported instead of means and SDs, suitable approximations will be used, as discussed in the *Cochrane Handbook*[38]. A standardized mean difference (SMD) and 99% confidence interval will be calculated for each study; a SMD >0 will indicate better overall training effectiveness. Where proportions of participants in each comparison group are reported for a particular outcome in an evaluation, this information will be collected. A relative risk (RR) and 99% confidence intervals will be calculated for each study. A RR >1.0 will indicate a higher proportion of participants with positive outcomes. Confidence levels of 99% will ensure conservative estimates of precision are obtained. If reporting and sample size allow, standard methods - depending on the approximate distribution of the data - will be used to transform medians and interquartile ranges (IQRs) to mean difference (MD) and SD, to allow visual inspection of estimates of effect. Where possible, these estimates will be included in SMD calculations for overall training effect.

#### *Dealing with missing data*

Corresponding authors of potentially included evaluations will be contacted, up to two times, where data are needed. If the data are not obtained and compromise the ability to include the evaluation in quantitative synthesis, it will be excluded from quantitative synthesis.

#### *Data synthesis*

General study characteristics will be presented in tabular format. Due to the anticipated methodological heterogeneity of potentially included evaluations (based on previous experience carrying out methodological systematic reviews), it is unlikely that data will be combined across evaluations. If this is the case, data will be described qualitatively in the text of the review. However, we will first assess for the suitability of meta-analysis, which will depend on the quantity of data and the homogeneity of studies according to methodology and content. If meta-analysis is possible, analyses will be conducted with the random effects model using the Review Manager software [39]. Any follow-up time for outcomes will be considered relevant, but only similar time points will be meta-analyzed; ‘similarity’ will need to be determined *post hoc* once study data are collected during the data extraction phase. Initially, all training programs will be considered together.

#### *Subgroup analysis*

If relevant data are reported and permit quantitative synthesis, the following subgroup analyses are planned: 1) modes of training delivery (that is, online, face-to-face), 2) primary role (that is, author, peer review, editor), 3) primary occupation of participant (that is, student (including medical residents), health practitioner, other health-related professional), 3) duration of training (that is, class, course, program), 4) credibility via institutional affiliation (that is, sponsored by academic institution, publishing house, industry, other), 5) setting (that is, individual versus group), or 6) associated cost (that is, cost versus no cost).

#### *Assessment of heterogeneity*

If there is any quantitatively aggregated data across included studies, we plan to measure the inconsistency of study results using the I^2^ heterogeneity statistic to determine the extent of variation in effect estimates that is due to heterogeneity rather than chance [38]. Heterogeneity will be determined by visual inspection of the forest plot and I^2^ statistics. For the interpretation of I^2^, a rough guide of low (0% to 25%), moderate (25% to 50%), substantial (50% to 75%), and considerable (75% to 100%) heterogeneity will be used [38]. Where considerable statistical heterogeneity exists (≥75%) data will not be pooled. Possible reasons for heterogeneity will be explored in sensitivity analyses; the pre-specified subgroup analyses, if feasible, will be examined to determine whether they provide possible reasons for any observed statistical heterogeneity. The variables outlined above for subgroup analyses will be considered statistically significant at *P* <0.01.

#### *Reporting biases*

Asymmetry of funnel plots is an established method for assessing the potential presence of publication bias, and others biases, in traditional systematic reviews of intervention effectiveness, subject to a sufficient number of included studies [38,40]. We will generate funnel plots and graphically explore the presence of asymmetry. If warranted we will evaluate asymmetry using statistical tools [40].

## Discussion

This project aims to provide evidence to help guide the journalological training of authors, peer reviewers, and editors, and the development of future training opportunities in this domain. While there is ample evidence that many members of these groups are not getting the necessary training needed to excel at their respective journalology-related tasks, little is known about the characteristics of existing training opportunities, including their effectiveness. The proposed systematic review will provide the evidence regarding the effectiveness of training, therefore giving potential trainees, course designers, and decision-makers evidence to help inform their choices and policies regarding the merits of a specific training opportunity or type of training.

We believe that the results of the proposed review will be of relevance to a wide variety of knowledge users, namely: authors, peer reviewers, and editors, as well as designers and teachers of training courses related to journalology, and decision-makers for continuing medical education (CME) and continuing professional development (CPD). Consumers of training (that is, potential trainees) will benefit by learning which types of training provide the most effective learning outcomes. This will empower them to make more informed choices regarding specific training, rather than making decisions based on word-of-mouth recommendations, academic affiliation, and other such unreliable methods. The designers of training will benefit by gaining access to a knowledge synthesis that outlines the characteristics of effective learning structures and environments. This will enable them to design better learning strategies and a better curriculum that takes into consideration the particularities of education in journalology. Finally, decision-makers will benefit by gaining an understanding of what works when choosing the type of training that will best benefit their organizations.

This review will provide the knowledge that is necessary for better educating authors, peer reviewers, and editors on how to reduce biomedical research waste by improving the quality and rigor in research reporting. Ultimately, the goal is to move closer to optimal reporting of health research, so that we can have full access to, and use of, the new knowledge coming from our investments in research.

## Abbreviations

AFP: American Family Physician; BMJ: British Medical Journal; CMAJ: Canadian Medical Association Journal; CME: Continuing medical education; COPE: Committee on Publication Ethics; CPD: Continuing professional development; EQUATOR: Enhancing the Quality and Transparency of Health Research; IQR: Interquartile range; JAMA: Journal of the American Medical Association; MD: Mean difference; RCT: Randomized controlled trial; RR: Relative risk; SD: Standard deviation; SMD: Standardized mean difference; WAME: World Association of Medical Editors.

## Competing interests

The authors declare that they have no competing interests.

## Authors’ contributions

JG drafted the manuscript; DM conceived of the study, participated in its design and coordination, and helped draft the manuscript; BS designed the search strategy; and CC, PHendry, DWC, PHébert, and AP all provided content expertise and participated in the design of the study. All authors read and approved the final manuscript.
